#  Congenital Cecal Duplication Cyst Mimicking as Intramural Mass

**DOI:** 10.21699/jns.v5i4.381

**Published:** 2016-10-10

**Authors:** Kamal Nain Rattan, Sunita Singh, Shruti Bansal, Megha Ralli, Ritika Vashisht

**Affiliations:** 1 Department of Pediatric Surgery, Pt. B.D. Sharma PGIMS Rohtak, Haryana; 2 Department of Pathology, Pt. B.D. Sharma PGIMS Rohtak, Haryana

**Dear Sir**

Cecal duplication cyst is one of the rarest forms of alimentary tract duplication.[1,2] Occurring intraluminally can mimic as intramural mass and intussusception. Thus it becomes quite daunting to make a specific diagnosis preoperatively. 

A 7-day-old male child with normal birth history and uneventful neonatal period was brought with complaints of bilious vomiting, abdominal distension and constipation. On examination, abdomen was soft with visible peristalsis; a lump was palpable in right iliac fossa. X-ray abdomen showed multiple air fluid levels. Ultrasonography abdomen revealed a mass in right iliac fossa. After adequate resuscitation, the child was taken up for surgery. On exploratory laparotomy, there was a firm intramural mass of size 3×3 cm palpable in ileocecal region causing obstruction with dilatation of whole of the small intestine proximal to the lesion and collapsed distal colon (Fig.1). Resection of the gut along with lesion was performed and ileo-colic anastomosis done. Grossly, a cystic globular structure was identified sharing a common wall with caecum and filled with mucoid material. Postoperative period was uneventful. The histopathological examination showed the cyst has all layers of large intestine, without any ectopic tissue, and shared a common wall with the caecum. 

**Figure:**
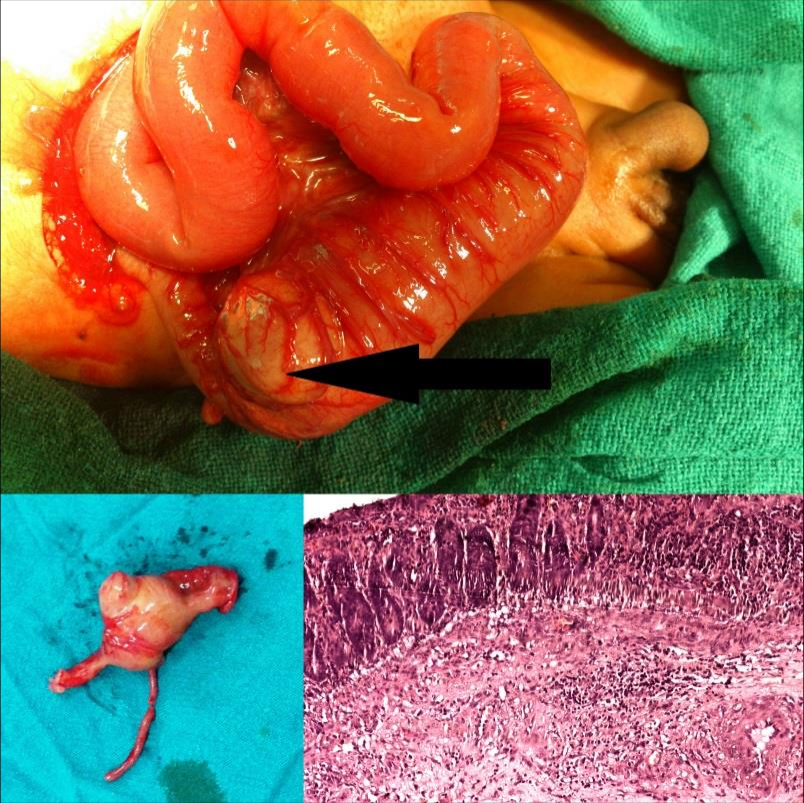
Figure 1: (Upper) Peroperative photograph showing an intraluminal cystic lesion at ileocecal region with dilated small intestine proximal to the lesion. (Lower-right) Photograph showing resected cystic lesion with dilated proximal fragment and narrowed distal fragment. (Lower-left) Photomicrograph showing the lining of cystic lesion (Hematoxylin & eosin stain, × 100).

Cecal duplications are exceedingly rare on literature search. [3-5] Cecal duplications can either simulate or lead to intussusceptions. [2] It can also present with features of intestinal obstruction. Ijaz et al [4] in and Keum et al [5] reported cases of cecal duplication presenting with acute intestinal obstruction. In present case, it was mimicking as intramural tumor mass. Cecal duplication cysts are although rare but should be kept in mind in neonates with intestinal obstruction.

## Footnotes

**Source of Support:** Nil

**Conflict of Interest:** None
